# *Potentilla anserina* reshapes soil-microbe networks in alpine meadow restoration

**DOI:** 10.3389/fmicb.2026.1890269

**Published:** 2026-08-11

**Authors:** Daobai Zhang, Xinying Han, Huakun Zhou, Junqiao Li, Meiling Jing

**Affiliations:** 1 College of Ecological Environment and Resources, Qinghai Minzu University, Xining, Qinghai, China; 2 Qinghai Provincial Key Laboratory of Restoration Ecology in Cold Region, Northwest Institute of Plateau Biology, Chinese Academy of Sciences, Xining, Qinghai, China; 3 Qinghai Provincial Key Laboratory of High Value Utilization of Characteristic Economic Plants, Xining, Qinghai, China; 4 Key Laboratory of Qinghai-Tibet Plateau Resource Chemistry and Ecological Environment Protection, National Ethnic Affairs Commission, Xining, Qinghai, China

**Keywords:** co-occurrence networks, ecosystem services, microbial assembly, *Potentilla anserinal*, rhizosphere effects

## Abstract

The formation of bare patches in degraded alpine meadows represents a critical bottleneck for ecological restoration. *Potentilla anserina* can rapidly cover exposed soil surfaces owing to its strong capacity for asexual reproduction. However, whether its secondary ecological functions extend beyond physical coverage remains unclear. In August 2024, rhizosphere and bulk soil samples were collected from both bare patches and *P. anserina*-colonized patches and analyzed via 16S rRNA and ITS high-throughput sequencing. The results revealed that the ameliorative effects of plants on soil physicochemical properties and microbial biomass extended beyond the rhizosphere, achieving overall optimization of the soil environment within patches. Analysis of community assembly processes based on βNTI revealed that colonization by plant synchronously increased the relative importance of environmental selection in both rhizosphere and bulk soil bacterial communities, indicating a global regulatory effect on bacteria. |βNTI| > 2 indicated deterministic assembly (environmental filtering), whereas |βNTI| < 2 reflected stochastic processes, which were further parsed by RC_bray: < −0.95 for homogenizing dispersal and >0.95 for dispersal limitation. In contrast, dispersal limitation was significantly greater only in the rhizosphere for fungal communities, indicating strict rhizosphere specificity. This divergent assembly process led to a simplified structure in bacterial communities, whereas fungal communities, through their unique ecological strategies, retained more original characteristics while acquiring a more complex and stable network structure. Network analysis identified an unclassified bacterium and a *Preussia* fungus as potential keystone taxa, which may drive soil regulation and organic matter decomposition, respectively, though functional validation is still required. This study elucidates the soil improvement and microbial regulatory mechanisms driven by *P. anserina* roots, highlights the divergent assembly processes between rhizosphere and bulk soil microbial communities, and provides theoretical support for the restoration of degraded alpine meadows. A key limitation is that this study was conducted at a single time point; seasonal and interannual dynamics may alter the observed patterns, and future work should incorporate temporal sampling and functional assays to validate the inferred keystone roles.

## 1 Introduction

The Qilian Mountains, located on the northeastern edge of the Qinghai–Tibetan Plateau, serve as critical ecological barriers in China. During the degradation process, bare patches form on the surface of the grassland (Lin et al., 2015). Without excessive human intervention, these patches can undergo natural recovery through ecological succession (Li et al., 2014). However, this process often takes decades or longer. In contrast, assisted restoration measures—such as reseeding, fertilization, or soil amendment—hold the potential to accelerate vegetation reestablishment and soil functional recovery. Therefore, elucidating the ecological roles of key microbial taxa in degraded grasslands is critical for developing effective assisted restoration strategies. In the natural recovery process of bare patches, some broad-leaved herbaceous plants serve as pioneer species that colonize first, among which *Potentilla anserina* is a typical and prominent pioneer plant.

*P. anserina* is a perennial herb characterized by creeping stolons and a dense fibrous root system. Its clonal growth capacity enables rapid lateral expansion across bare patches, which reduces surface runoff and soil erosion. Concurrently, the extensive root architecture enhances soil aggregation and nutrient availability, thereby creating favorable edaphic conditions for the re-establishment of later-successional species.

Plant roots influence soil microbial communities through multiple pathways, including root turnover, litter inputs, the release of root exudates, symbiotic associations such as mycorrhizae, and competition for available nutrients in the soil (Philippot et al., 2013; Trivedi et al., 2020; Ling et al., 2022; Delory et al., 2024; Wardle et al., 2004). Within the rhizosphere, the outcomes of plant–microbe interactions represent a continuum of ecological trade-offs rather than a simple dichotomy of beneficial versus pathogenic effects. Microbes that enhance plant growth by facilitating nutrient acquisition or regulating phytohormonal activity can, under resource limitation or competitive suppression, become neutral or even detrimental (Santoyo et al., 2021). Conversely, pathogenicity is often context-dependent: opportunistic pathogens may remain asymptomatic until plant defense thresholds are breached by environmental stress. Consequently, the net effect of any microbial taxon on plant performance depends on the balance between its growth-promoting and disease-suppressing capacities, which are modulated by soil fertility, plant genotype, and environmental stress (Ortíz-Castro et al., 2009; Luo et al., 2025). Soil microorganisms function as key decomposers in terrestrial ecosystems (Zechmeister-Boltenstern et al., 2015), driving essential biogeochemical processes such as the cycling of carbon (Li et al., 2019), nitrogen (Spohn and Kuzyakov, 2013), and phosphorus (Trivedi et al., 2013). It has been suggested that biogeochemical cycling processes in underground ecosystems are associated with plants. Microbial community composition is closely linked to plant functional traits that reflect ecological strategies (Bardgett et al., 2021). For example, fast-growing plant species tend to contribute more labile litter and root exudates, which in turn favor microbial taxa associated with rapid nutrient turnover, particularly bacteria.

Through its biological characteristics, *P. anserina* indirectly or directly participates in and contributes to ecosystem services such as carbon sequestration and water conservation. Its adaptability helps enhance the resilience of ecosystems to global change (Guan et al., 2024; Zhou et al., 2024). As a dominant species in specific habitats, *P. anserina* provides habitats and food sources for other organisms, influencing plant community structure, soil physical and chemical properties, and microbial communities. It is crucial for maintaining biodiversity and ecosystem stability (Liu et al., 2021). The rapid growth and clonal reproduction capabilities of *P. anserina* make it an ideal species for the ecological restoration of degraded lands, as it is capable of forming vegetation cover in damaged areas such as mining sites (Ranđelović et al., 2024). However, the mechanisms by which this plant affects rhizosphere soil following its establishment remain unclear. Consequently, a systematic and in-depth investigation of the interactions between *P. anserina* and soil microorganisms underground is beneficial for providing theoretical support for its use in the restoration of degraded grasslands. This research also offers significant strategies and insights for addressing the ecological challenges faced by the Qinghai-Tibetan Plateau in the context of global change.

Here, we investigated how *P. anserina* affects community composition in the rhizosphere of the Qinghai-Tibetan Plateau. We aimed to (i) reveal the distinct assembly mechanisms of bacterial and fungal communities in the rhizosphere and (ii) evaluate how plant-driven environmental filtering influences the network architecture of microbial communities. This study addresses two key questions regarding the “rhizosphere effect” of *P. anserina*: (i) Does the plant reshape the rhizosphere microenvironment through its root activities, and if so, does this effect differentially regulate the assembly processes of bacterial versus fungal communities? (ii) Specifically, does enhanced deterministic selection in bacterial communities lead to simpler, more specialized network structures, and does reduced stochasticity in fungal communities promote the establishment of specific symbiotic or interactive relationships?

## 2 Materials and methods

### 2.1 Site description and soil sampling

This research was conducted in the Qilian Mountains (37°56′43″ N, 100°13′08″ E), which are located in Qinghai Province, China. The altitude is approximately 3578.12 m. The area has a temperate continental arid climate with an average annual temperature of 1.4 °C. The mean annual precipitation is 415.0 mm, occurring primarily between June and September, and there is no absolute frost-free period. Light-energy resources are abundant, with 2,829 h of sunshine annually and intense solar radiation. The accumulated temperature above 0 °C is 1,658.0 °C, and the mean annual evaporation is 1,162.3 mm. The area encompasses a mosaic of complex habitats with rich vegetation dominated largely by cold-region endemics.

**FIGURE 1:**
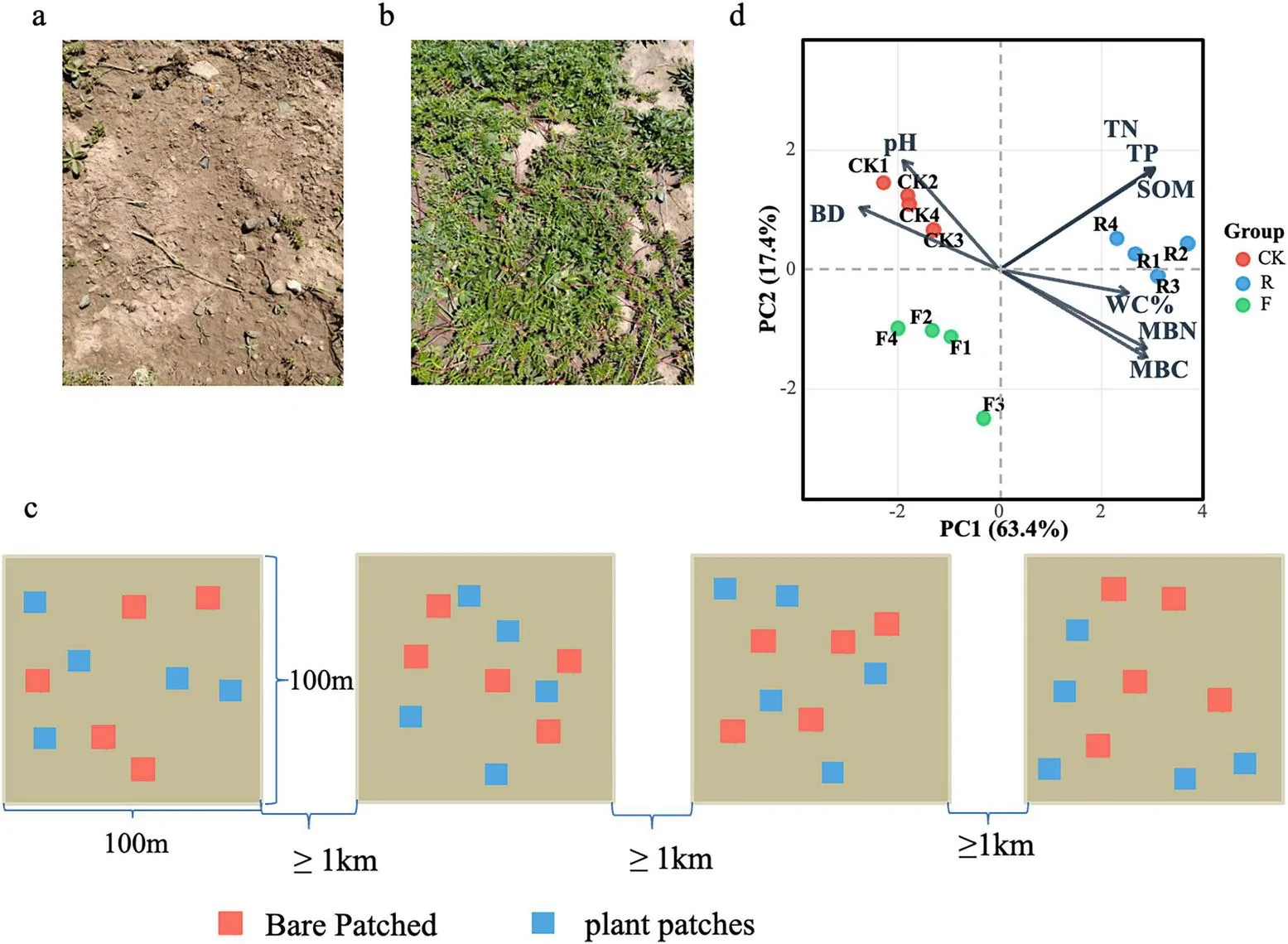
Completely degraded bare patches **(a)**, bare patches covered with Potentilla anserina **(b)**, principal component analysis (PCA) biplot **(c)** and Sample selection method **(d)**. WC%, water content; BD, bulk density; SOM, soil organic matter; TN, total nitrogen; TP, total phosphorus; MBC, microbial biomass carbon; MBN, microbial biomass nitrogen.

In August 2024, we selected moderately degraded alpine meadow as the experimental site for field surveys and soil sampling. We established four replicate sampling zones, each covering an area of approximately 1 hm^2^, with the distance between adjacent zones being no less than 1 km. Within each sampling zone, we set up two types of sampling points: (1) bare patches with vegetation cover close to 0, occasionally scattered with broad-leaved weeds, and characterized by loose, exposed soil (Figure 1a); and (2) plant patches where only *P. anserina* was distributed, with vegetation cover ranging from 60% to 80% (Figure 1b). At each bare-patch sampling point, we collected five bare-patch soil subsamples. The distance between adjacent subsample bare patches was ≥20 m, and the diameter of each subsample bare patch was ≥1 m. These five subsamples were thoroughly mixed into one composite sample, yielding a total of four independent soil samples, labeled CK. At each plant-patch sampling point, we excavated multiple healthy plants of uniform size and growth status, and used the shaking method to obtain five rhizosphere and five non-rhizosphere soil subsamples, each of which was separately composited into one sample. This resulted in four independent rhizosphere soil samples and four independent non-rhizosphere soil samples, labeled R and F, respectively. In total, 12 soil samples were obtained from the above experiment (Figure 1c). Any visible stones or root fragments were manually removed. Each sample was divided into three portions: one portion was stored at −80 °C for soil microbial sequencing; one portion was passed through a 2 mm sieve and stored at 4 °C for determination of soil microbial biomass indices; and the remaining portion was air-dried, with roots and gravel removed, ground, and sieved for measurement of soil physicochemical properties.

### 2.2 Soil property analyses

A glass electrode was used to determine the soil pH in a 1:2.5 soil:water (w:v) suspension. SOC was quantified via the dichromate oxidation method, whereas total nitrogen (TN) was measured via Kjeldahl digestion (Black, n.d.). The extraction of soil total P (TP) was performed via HF–HClO_4_, followed by determination via the molybdenum-blue method. The soil organic matter content was determined via the K2Cr2O7-H2SO4 oxidation method. Microbial biomass carbon was quantified via the chloroform fumigation–extraction approach (Vance et al., 1987). MBC was calculated as the difference in carbon concentration between fumigated and non-fumigated samples, divided by a conversion factor of 0.38 (Joergensen, 1996). Both fumigated and non-fumigated soil samples were extracted with 0.05 M K_2_SO_4_ at a soil-to-solution ratio of 1:4 (w:v), and the extracts were subsequently analyzed via a Multi N/C 3100 TOC/TN analyzer. Microbial biomass phosphorus (MBP) was assayed via chloroform fumigation–NaHCO_3_ extraction, colorimetric Pi determination and external Pi correction (Smirnova et al., 2021). The cutting ring method was employed to measure the soil bulk density (BD), and the WC% was quantified via the oven drying method.

### 2.3 DNA extraction and sequencing

DNA was extracted via the E.Z.N.A.^®^ Soil DNA Kit (Norcross, GA, United States) and submitted to “Shanghai Majorbio Bio-Pharm Technology Co., Ltd.” for paired-end sequencing (2 × 250 bp) on the “Illumina NovaSeq 6000 platform.” The V3-V4 hypervariable region of the bacterial 16S rRNA gene was amplified via the primers “338F (5′-ACTCCTACGGGAGGCAGCA-3′)” and “806R (5′-GGACTACHVGGGTWTCTAAT-3′,” whereas the fungal ITS2 region was amplified via the primers “ITS3F (5′-GCATCGATGAAGAACGCAGC-3′)” and “ITS4R (5′-TCCTCCGCTTATTGATATGC-3′).” Sequencing data were processed via the QIIME2 platform (Magoč and Salzberg, 2011), where operational taxonomic units (OTUs) were clustered at 97% sequence similarity, and taxonomic classification was performed via comparison with the SILVA (for bacteria) and UNITE (for fungi) databases (Stackebrandt and Goebel, 1994; Edgar, 2013).

### 2.4 Statistics and data analysis

Statistical comparisons of soil physicochemical parameters, α-diversity indices, and network topological characteristics were conducted via the Kruskal–Wallis test in R 4.5.1. Principal component analysis (PCA) visualized soil parameter differences among zones, and community dissimilarities were ordinated via PCoA on Bray–Curtis distances. Significance was tested via PERMANOVA (9,999 permutations) with Benjamini–Hochberg correction.

Redundancy analysis (RDA) linked microbial communities to soil variables (Borcard and Legendre, 2002). Co-occurrence networks were built from Spearman correlations (|r| > 0.8, FDR-corrected *P* < 0.01) and compared against 1,000 random networks (Steinhauser et al., 2008). Nodes were categorized into four topological roles on the basis of within-module connectivity (Zi) and among-module connectivity (Pi) (Olesen et al., 2007).

Operational taxonomic units with a relative abundance >0.01% in at least one sample were retained to reduce complexity (Jeewani et al., 2021). The igraph package in R (Csardi and Nepusz, n.d.) was used to extract subnetworks, and 1000 Erdos–Renyi random networks were constructed for comparison (Erds and Rényi, n.d.). The networks were visualized via Gephi (Bastian et al., 2009). and subnetworks were extracted with the igraph package in R.

Community assembly mechanisms were assessed via null model approaches on the basis of the nearest taxon index (NTI) and β-nearest taxon index (βNTI). Values of |βNTI| greater than 2 were interpreted as evidence of deterministic processes, whereas values less than 2 suggested stochastic influences (Stegen et al., 2013). For cases where |βNTI| was less than 2, the Raup–Crick metric derived from Bray–Curtis dissimilarity (RC_bray) was further applied to distinguish among stochastic components, including homogenizing dispersal (RC_bray < −0.95), dispersal limitation (RC_bray > 0.95), and undominated processes (|RC_bray| < 0.95). NTI was computed via the picante package in R, whereas βNTI was estimated via Phylocom version 4.1 (Hardy, 2008).

## 3 Results

### 3.1 Enrichment of nutrients in rhizosphere soil compared with non-rhizosphere soil

Soil physicochemical analysis indicated that the BD of the rhizosphere soil samples (R) was significantly lower than that of the non-rhizosphere soil samples (F) and the control samples (CK). Compared with those of CK, the pH values of R and F significantly decreased. The SOM, TN and TP contents in the R samples were significantly greater than those in the CK samples, whereas those in the F samples were significantly lower than those in the CK samples. Notably, both the MBC and MBN in R and F were significantly greater than those in CK (Supplementary Table 1). Principal component analysis (PCA) of the soil physicochemical properties revealed three major components (PC1–PC3), which together accounted for 89.25% of the total variance (Supplementary Table 2). The first component (PC1), explaining 63.36% of the variance, was strongly and positively associated (loadings > 0.80) with soil organic matter (SOM), total nitrogen (TN), total phosphorus (TP), microbial biomass carbon (MBC), and microbial biomass nitrogen (MBN) (Figure 1d and Supplementary Table 2). This component represented a gradient of soil nutrient changes driven by organic matter, with nutrients being more enriched in the rhizosphere soil than in the non-rhizosphere soil (Supplementary Table 1). PC2 explained an additional 17.41% of the variance (Supplementary Table 2). These PCA results collectively indicate that nutrient enrichment—particularly the buildup of organic matter, nitrogen, and phosphorus—constitutes the primary ecological driver differentiating the restored patches from the degraded bare soil. Distinct environmental differences were observed between R and F, as well as between R and CK along PC1. Significant differences were evident between F and CK along PC2 (Supplementary Table 3).

### 3.2 Rhizosphere and non-rhizosphere soil microorganism diversity and community structure

Most of the OTUs were present across the three zones (Supplementary Figure 2). Specifically, 28.60% of the bacterial OTUs were common to all zones, whereas only 16.09% of the fungal OTUs were shared across the same areas. Principal coordinate analysis (PCoA) revealed clear differences in community composition among the three sample groups (Supplementary Figure 3). The bacterial assemblages from the CK samples showed pronounced separation along the first principal coordinate (PCo1), which accounted for 31.95% of the variation. Similarly, fungal communities were primarily differentiated along PCo1, explaining 21.27% of the variance. The R samples and F samples clustered separately on PC2 for bacteria (with 22.13% explanatory power) and PC2 for fungi (with 18.62% explanatory power), respectively. Supplementary Table 4 supports this (*P* < 0.01). The higher bacterial OTU overlap (28.60%) relative to fungal overlap (16.09%) may reflect greater niche conservatism among bacteria, which can persist across heterogeneous soil microsites via dormancy and rapid growth responses. In contrast, the lower fungal overlap suggests stronger community turnover driven by niche differentiation and dispersal limitation, particularly in the rhizosphere where root-specific fungal associations are selectively enriched. Regardless of the bacterial or fungal community, spatial clustering demonstrated a high degree of variability, indicating strong heterogeneity among different samples.

**FIGURE 2:**
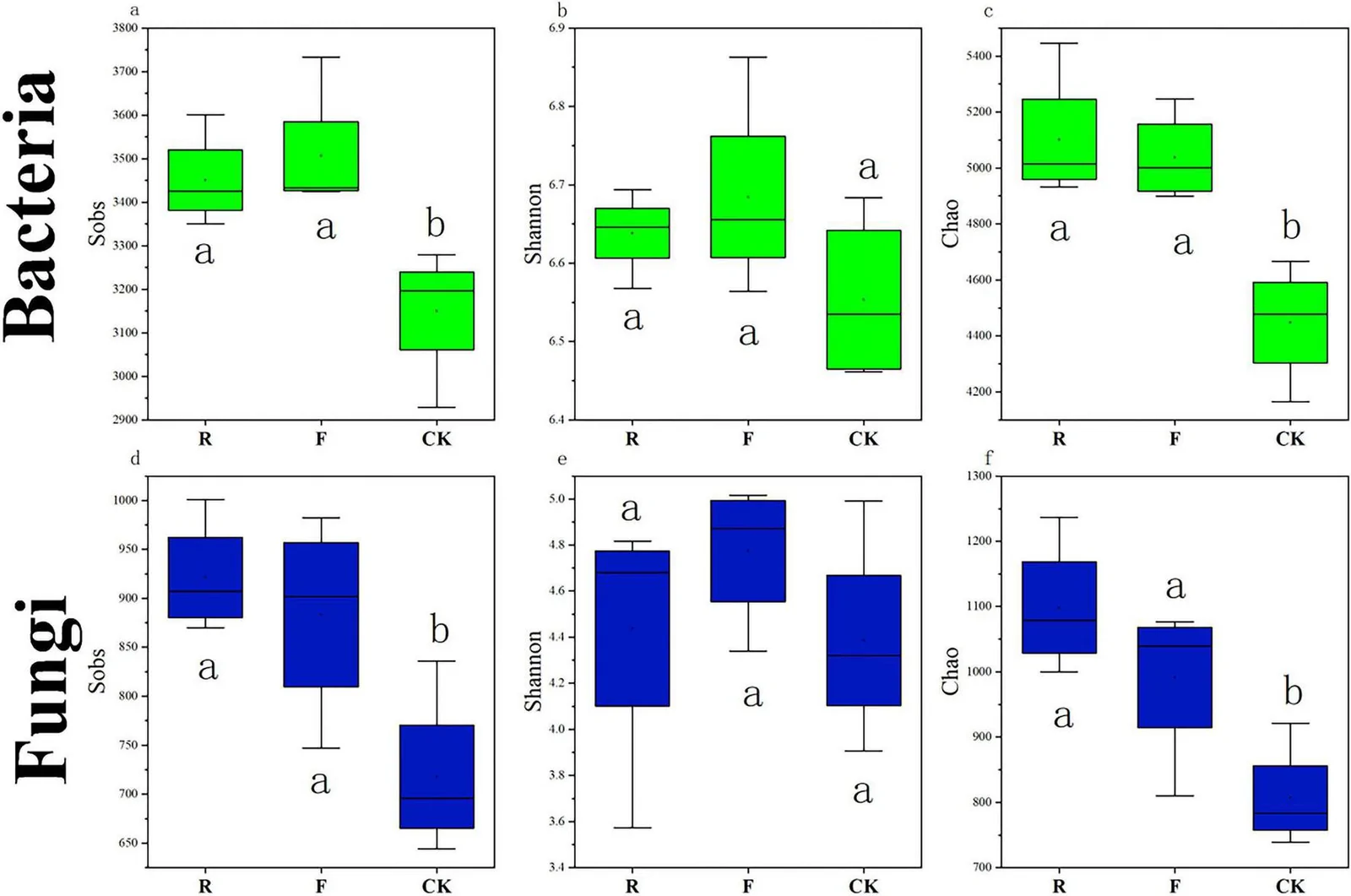
Bacterial alpha diversity indices: Sobs **(a)**, Shannon **(b)**, and Chao **(c)**; fungal alpha diversity indices: Sobs **(d)**, Shannon **(e)**, and Chao **(f)**. Different letters indicate significant differences (*P* < 0.05) between zones.

The α diversity indices (Sobs index, Shannon index, and Chao index) presented parallel trends, with lower values in the CK treatment than in the R and F treatments (Figure 2). For both the bacterial and fungal communities, the Sobs index and Chao index were significantly greater in R and F than in CK (*P* < 0.05). However, no significant differences were observed in the Shannon index among the three groups.

**FIGURE 3:**
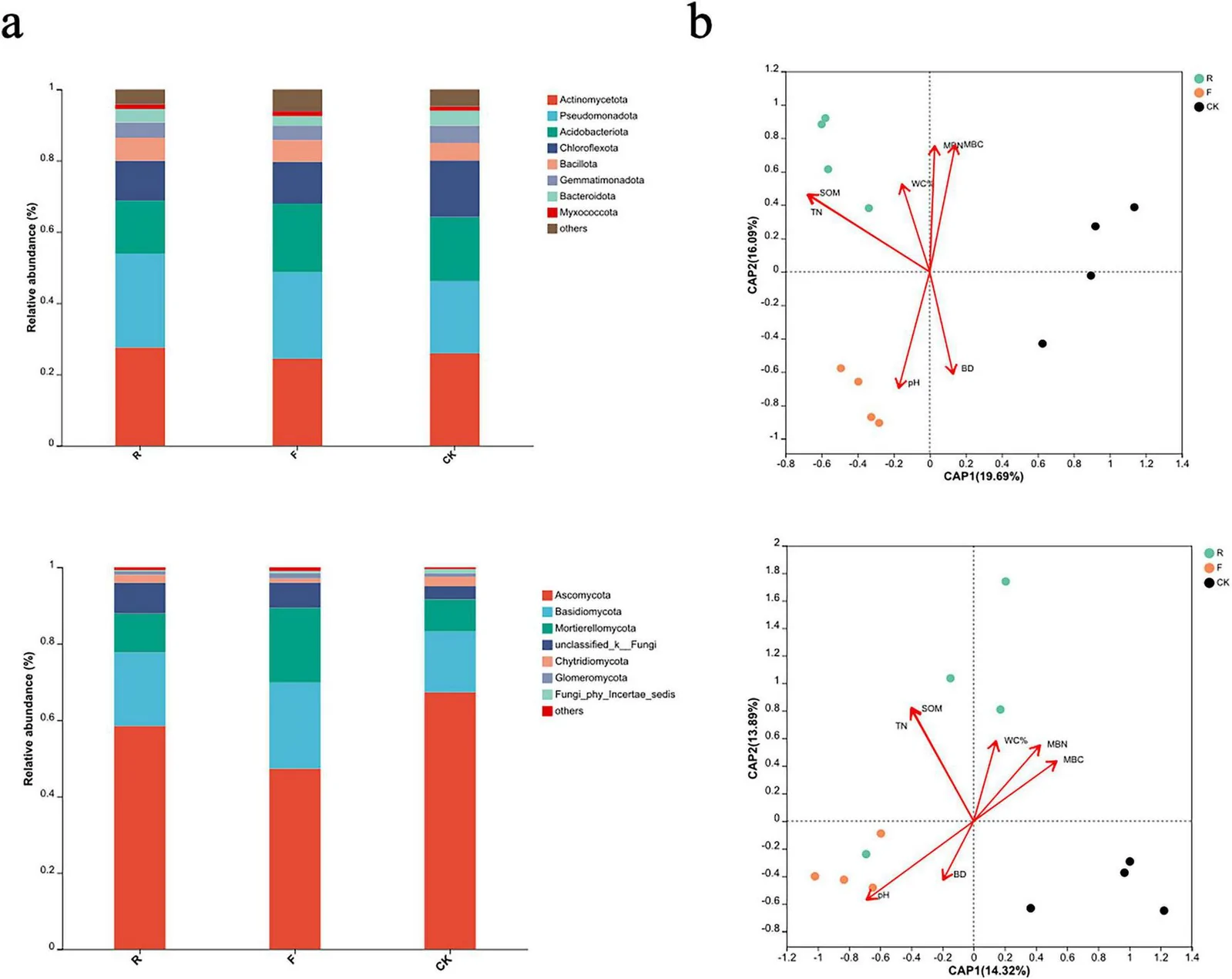
Relative abundances of the dominant bacterial and fungal phyla **(a)** and the redundancy analysis (RDA) results illustrating the associations between the soil microbial communities at the operational taxonomic unit (OTU) level and the environmental variables **(b)**.

The bacterial community composition was dominated by Actinomycetota (19.7%–30.0%), Pseudomonadota (17.2%–30.1%) and Acidobacteriota (9.9%–26.6%), followed by Chloroflexota (8.1%–19.6%) and Bacillota (3.1%–9.0%), accounting for 83.0%–87.8% of the sequences (Figure 3 and S4). The fungal community was dominated by Ascomycota (32.1–75.9%), followed by Basidiomycota (8.1–40.0%), Mortierellomycota (4.6–24.9%) and unclassified_k_Fungi (2.0–17.0%), collectively representing 94.64–97.11% of the sequences (Figure 3a and Supplementary Figure 4).

Distance-based redundancy analysis (db-RDA) indicated that the environmental factors influencing the bacterial and fungal communities differed markedly (Figure 3b). The first two axes explained 35.78% of the variation in bacterial community composition, which was driven primarily by microbial biomass carbon (MBC) and microbial biomass nitrogen (MBN), whereas they accounted for 28.21% of the fungal variation, which was largely associated with TN and SOM. The Mantel test results supported these findings, revealing a strong correlation between bacterial communities and soil variables (*r* = 0.608, *P* = 0.002), and fungi presented a relatively weak relationship with soil factors (*r* = 0.316, *P* = 0.015). Biologically, the stronger bacterial–soil coupling suggests that bacteria are more responsive to the edaphic changes induced by *P. anserina*—such as shifts in pH, nutrient availability, and microbial biomass—than fungi are.

### 3.3 Co-occurrence networks of communities

**FIGURE 4:**
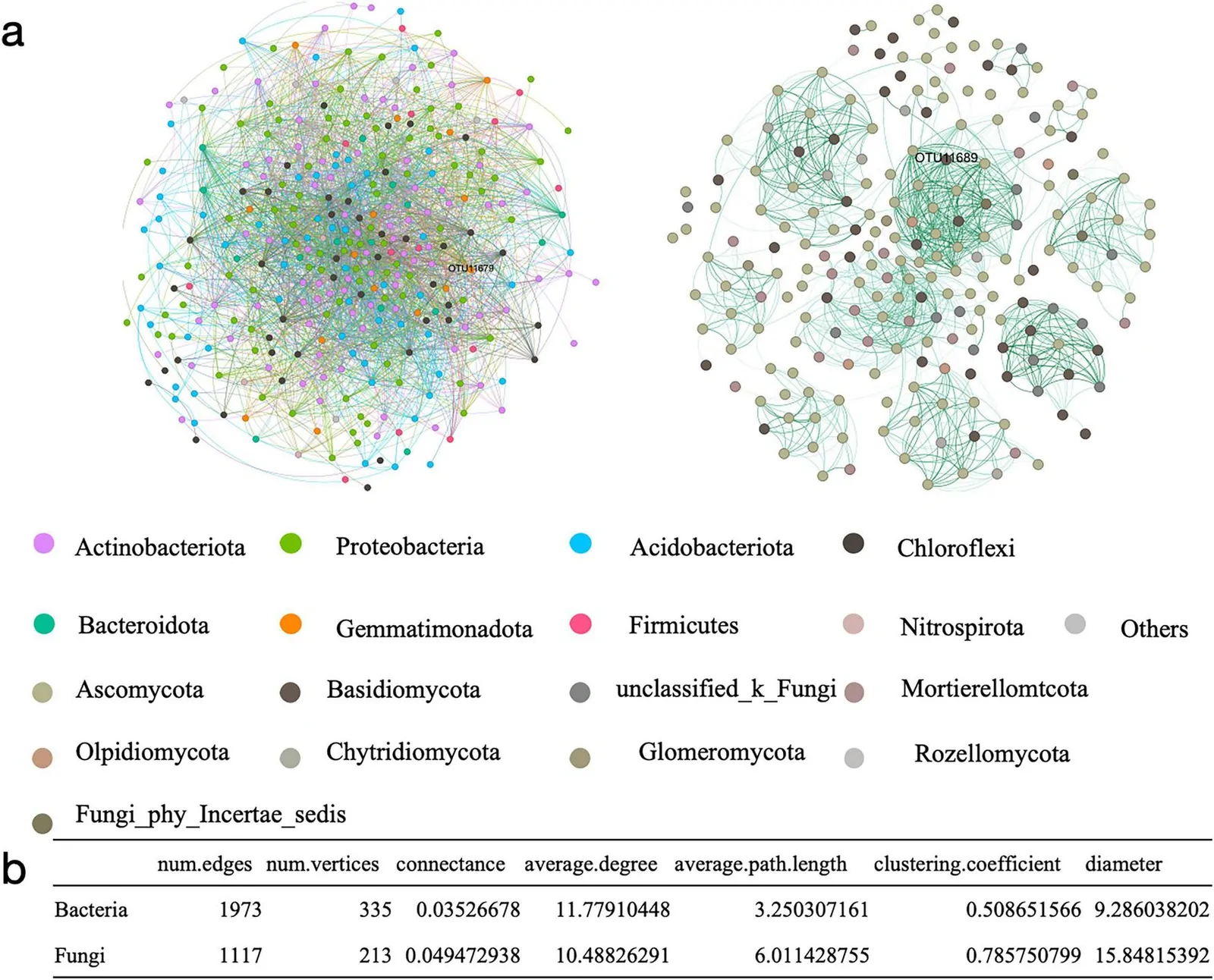
Co-occurrence network of microbiome operational taxonomic units (OTUs) inferred from correlation analysis across all sampling sites **(a)**. Zone-specific subnetworks for bacteria (Supplementary Figure 5) and fungi (Supplementary Figure 6) were also generated independently. Nodes are colored by phylum, and the legend indicates the dominant phyla ranked by relative abundance. The network topological characteristics of the bacterial and fungal communities are presented in panel **(b)**. The OTUs highlighted in the networks correspond to keystone taxa.

Co-occurrence networks for bacterial and fungal communities were generated at the OTU level (Figure 4a), along with subnetworks specific to each zone (Supplementary Figures 5, 6). Comparative analysis of the overall networks revealed distinct structural properties between bacteria and fungi. The bacterial network, comprising 335 nodes and 1,973 edges, displayed greater structural complexity, as indicated by a higher average degree (11.78 vs. 10.49). However, compared with the fungal network, the fungal network presented a shorter average path length and a lower clustering coefficient (Figure 4b). Keystone species analysis further identified potential keystone taxa: a key bacterial module hub belonging to the family Gemmatimonadaceae (phylum Gemmatimonadota), whereas only one fungal module hub, affiliated with the order Agaricales (phylum Basidiomycota), was detected. Because these inferences are based solely on network topology, we designate them as potential keystone taxa pending functional validation. In terms of bacteria, the module hub with the highest degree in R belonged to Oxalobacteraceae (Proteobacteria), whereas in F, it was IS-44 (Proteobacteria) and Micropepsaceae (Proteobacteria), and in CK, it was Nocardioides (Actinobacteriota) (Supplementary Figure 5). In terms of fungi, the greatest degree of fungi in R belonged to Clavariaceae (Basidiomycota), whereas in F, it was Mortierella (Mortierellomycota), and in CK, it was Olpidiaceae (Olpidiomycota) (Supplementary Figure 6).

### 3.4 Assembly processes of microbiome communities

**FIGURE 5:**
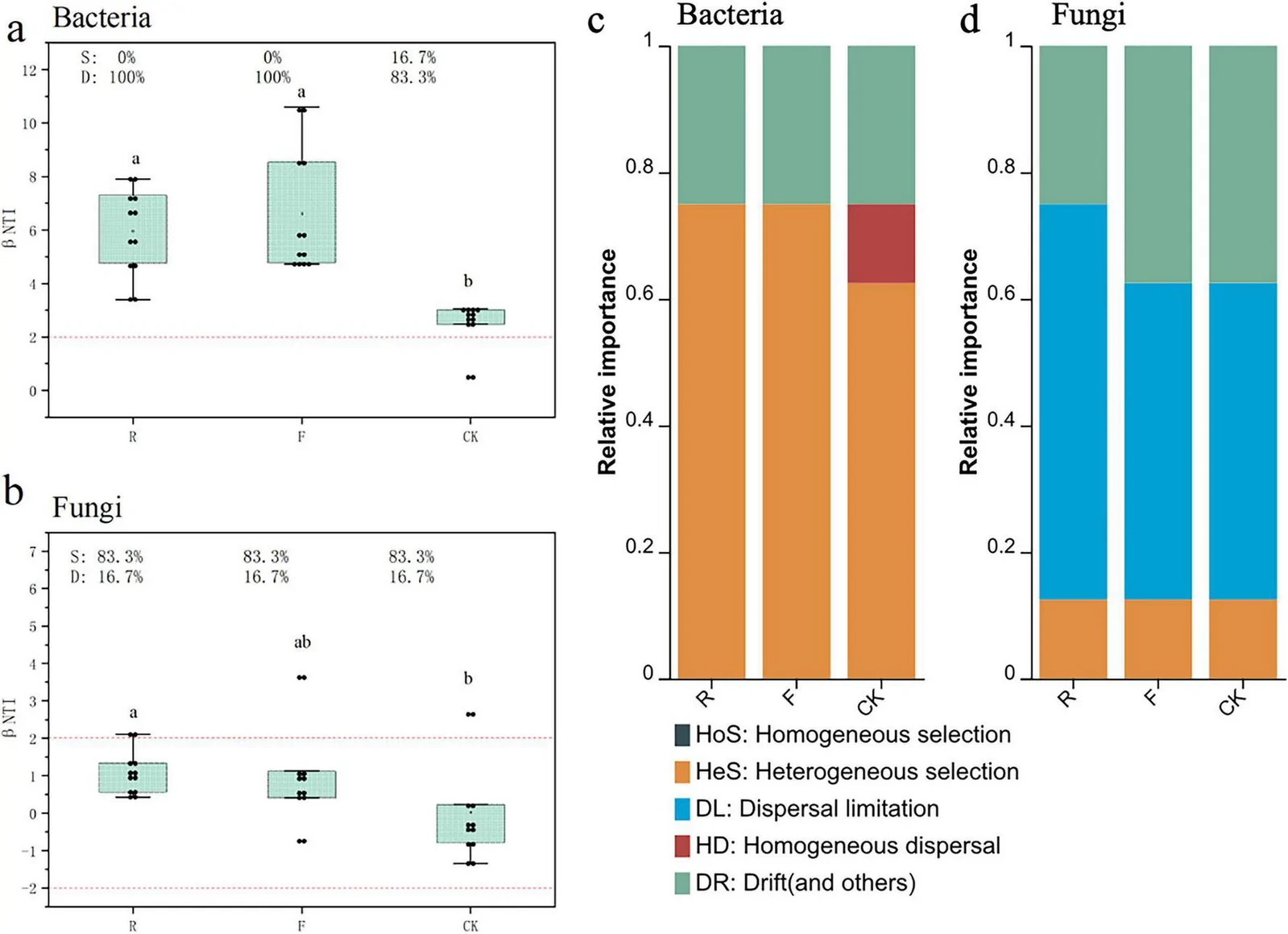
Boxplots illustrating βNTI values along the gradient **(a,b)**, together with the proportional contributions (%) of deterministic and stochastic mechanisms to community turnover across the entire study area **(c,d)**. Different letters indicate significant differences (*P* < 0.05) between zones.

The bacterial communities exhibited strong phylogenetic clustering, with 94.4% of the βNTI values exceeding the threshold of 2, indicating the dominance of deterministic assembly processes and suggesting a dominant role of deterministic processes, particularly variable selection Figures 5a, c. In contrast, most fungal βNTI values (83.3%) fell within the stochastic interval, indicating that stochastic processes prevailed. Among these, dispersal limitation accounted for the largest proportion (50.0%–62.5%), followed by undominated processes (25.0%–37.5%) (Figures 5b, d).

## 4 Discussion

### 4.1 The establishment of P. anserina explains the differences in the soil microbial communities

Our research demonstrated that the establishment of *P. anserina* is the primary driver behind the fundamental differences observed in the surrounding soil microbial structure. The distinct differences in microbial communities between *P. anserina* soils (both R and F) and the control (CK) soils suggest that plant establishment plays a critical role in shaping soil microbial assemblages, which aligns with findings from other studies showing that different plant species harbor distinct rhizosphere microbial communities. For different plant species, the distance and scope of the rhizosphere effect are not fixed. In this study, *P. anserina* L. caused differences in the physicochemical properties of bare patch soil before and after coverage, which was confirmed by the results of the RDA. This change then affected the microbial communities, which is consistent with the findings of previous studies (Ling et al., 2022; Sun, 2024). In alpine grasslands, the content of soil organic matter and pH are crucial on the Qinghai–Tibetan Plateau, as they directly determine the evolutionary degree of communities and the survival of plants. Some microorganisms, such as plants, may have greater adaptability to low temperatures or nutrient stress, thereby gaining a competitive advantage in the rhizosphere environment. Traditional views hold that the rhizosphere effect primarily occurs within a few centimeters from the root surface outward (Liu et al., 2021). The significantly greater contents of microbial biomass carbon (MBC) and nitrogen (MBN) in the F treatment than in the CK treatment (Table 1 and Supplementary Figure 1) suggest that the influence of *P. anserina* roots may extend beyond the immediate rhizosphere. However, we cannot exclude the possibility that this pattern is instead attributable to the lateral spread of fine roots and mycorrhizal hyphae into the bulk soil fraction, or to root exudate diffusion and nutrient mobilization processes. Future fine-scale spatial sampling would be required to verify the precise spatial extent of these effects. Additionally, research has indicated that fungal communities vary with season and that the levels of nitrogen, phosphorus, and potassium in the soil are highly correlated with specific fungal groups and their distributions (Wang, 2021). This finding is consistent with our findings, collectively demonstrating that the root system of *P. anserina* can indirectly influence a broader range of microbial communities by affecting these nutrients.

**TABLE 1:** Mantel tests were performed to examine the relationships between the soil microbial communities (based on Bray–Curtis dissimilarity) and the measured soil environmental variables.

Microbial community	Mantel test
	Soil factors
Bacterial community	0.608 (*P* = 0.002)
Fungal community	0.316 (*P* = 0.015)

### 4.2 How does it affect the assembly processes of bacteria and fungi?

Investigating the soil microbial communities before and after the establishment of P. anserina in the bare patches revealed significant reshaping of the communities. We explored the different responses of these networks to environmental factors and assembly processes, highlighting the significant role of the rhizosphere effect in altering network topology. These results highlight the ecological complexity of the region and are consistent with findings from previous studies (Wu et al., 2025). However, as a plant species with distinctive characteristics from those of the Qinghai-Tibetan Plateau region, the effects of *P. anserina* differ considerably from those of other plant species.

Plants strongly influence bacterial networks, echoing research results on the role of plants in shaping bacterial community composition (Xiong et al., 2021; Tomazelli et al., 2024). This influence is consistent with our hypothesis. The observed decrease in bacterial community connectivity suggests a tendency toward structural simplification, which may reflect either genuine homogenization—driven by the selective enrichment of functionally similar taxa under the plant’s edaphic filter. We cannot conclusively distinguish between these alternatives without additional temporal or spatial replication, but the pattern is consistent with the idea that strong environmental selection can narrow the range of coexisting bacterial strategies.

The microbial networks in the degraded alpine meadows of the Qilian Mountains were studied, and the impacts of assembly processes and microbial diversity on these networks from relevant research were considered (Li et al., 2020; Zhang et al., 2022). The stability of fungi may stem from their unique survival strategies and physiological traits (Barnard et al., 2013). In fungal communities, the relative importance of dispersal limitation is greater than that in bacterial communities. This is primarily attributable to the fact that fungi rely on hyphal growth for spatial expansion, making their colonization processes more prone to “priority effects”—a phenomenon in which early-arriving species preemptively occupy niches and exclude later arrivals (Vannette and Fukami, 2014; Fukami, 2015). Such priority effects amplify the contribution of dispersal limitation while simultaneously narrowing the scope for environmental selection. In contrast, bacteria are characterized by small cell sizes, and some taxa employ flagellar motility, enabling effective dispersal along water films on root surfaces (Powell et al., 2015). This greater capacity to overcome spatial barriers makes it difficult to establish stable priority effects, and consequently, dispersal limitation carries a relatively lower weight in bacterial community assembly. Which is consistent with our initial hypothesis. The observed βNTI patterns further indicate that historical contingency and phylogenetic constraints play important roles in structuring fungal networks (Yuan et al., 2018; Feng et al., 2018). The relative importance of dispersal limitation was markedly greater in R than in F and CK. This pattern is likely driven by the highly differentiated root architecture of *P. anserina*, where distinct root components diverge considerably in terms of physical morphology and physiological function (Hinsinger et al., 2009). This structural differentiation generates intrinsic physical barriers and pronounced microspatial heterogeneity on the root surface. Constrained by their relatively larger spore or hyphal dimensions and the additional obstruction posed by mucilaginous root exudates, fungi experience substantially reduced spatial dispersal efficiency, thereby increasing the relative importance of dispersal limitation (Philippot et al., 2013).

The bacterial community assembly mechanisms did not significantly differ between R and F. Colonization by *P. anserina* synchronously increased the relative importance of environmental selection in both compartments, a pattern consistent with stronger environmental filtering under the plant’s edaphic influence (Stegen et al., 2013). However, this association does not establish causality: we cannot rule out confounding factors such as pre-existing soil heterogeneity or temporal legacy effects. It can thus be inferred that the observed shifts in bacterial community composition are correlated with colonization at the whole-soil scale rather than being spatially confined to the rhizosphere. In marked contrast, dispersal limitation was significantly greater only for fungal communities in the rhizosphere (Hinsinger et al., 2009). This pattern is consistent with the previously described microspatial heterogeneity generated by root architectural differentiation and displays a strict rhizosphere-specific characteristic.

### 4.3 Environmental factors jointly shape the structure of microbiome networks

Our study of microbial networks in degraded alpine meadows of the Qilian Mountains demonstrated that *P. anserina* can substantially modify the network architecture of both microbiome communities. In addition, our research examined how microbial diversity and community assembly processes contribute to the organization and stability of these networks.

**FIGURE 6:**
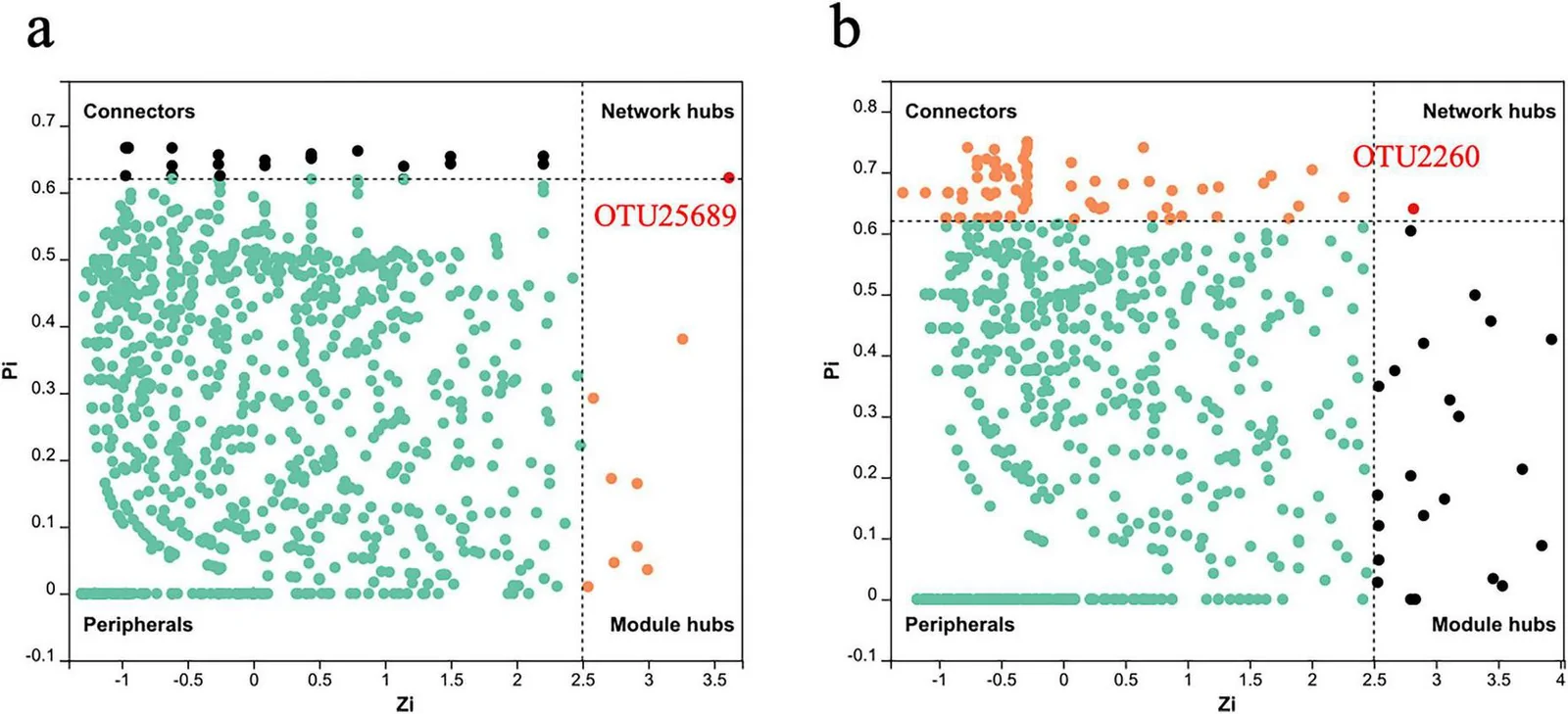
Zi–Pi plots showing the distribution of bacterial **(a)** and fungal **(b)** operational taxonomic units (OTUs) on the basis of their topological roles in the networks. Each symbol represents an OTU in the network.

Prioritizing dominant taxa allows for a clearer understanding of core network dynamics; however, this strategy entails a trade-off: interactions among rare taxa, which might be resolved via deeper sequencing, risk being missed (Jiao et al., 2017). In essence, this trade-off reflects a balance between analytical rigor and the possible loss of ecological detail. On the basis of the *Zi–Pi* framework (Figure 6; Olesen et al., 2007). However, the precise taxonomic identity and functional attributes of OTU 25689 remain unclear. It is inferred that this bacterium functions as a network hub, although the absence of cultivation-based or metagenomic functional assays prevents us from confirming its keystone role experimentally. As such, it may contribute to regulating and strengthening microbial interactions within bare patch environments colonized by *P. anserina*, potentially playing an important role in the species’ capacity to facilitate ecosystem recovery. In the fungal network, *Preussia* (OTU 2260), which serves as a network hub, had a significant effect on the stability of cooccurring bacterial networks. *Preussia* is a genus of filamentous, saprotrophic fungi that are commonly found in soil and on decaying plant matter, where they play a key role in decomposing complex organic material (Dodson and Field, 2018). During the succession of alpine degraded grasslands, following the collapse of partial meadow vegetation layers, many dead plant roots remain in the bare patches. Microorganisms with organic matter decomposition functions can degrade complex lignin and cellulose in dead roots through physical actions (invasive growth) and chemical degradation (enzyme secretion), thereby improving the soil nutrient cycling system and structure. In the long term, this decomposition produces a large amount of carbon sources and energy, maintaining a vast decomposer network in the soil, which helps to suppress the outbreak of soil-borne pathogens (Hu et al., 2018). This is sufficient to demonstrate that *P. anserina* is far from being a “simple plant” that merely increases ground cover to achieve superficial grassland restoration.

In summary, the observed shifts in bacterial community composition associated with *P. anserina* colonization are not spatially confined to the rhizosphere but rather occur at the whole-soil scale: the increased relative importance of environmental filtering in both R and F is consistent with plant-driven edaphic changes, though causality cannot be established without controlled experiments. In contrast, fungal community shifts display strict rhizosphere specificity, a pattern consistent with priority effects mediated by spatial barriers that restrict the accumulation of specific fungal taxa on root surfaces. These divergent assembly patterns are correlational: they describe observed associations between plant presence and microbial community structure but do not demonstrate causal links to ecosystem function. Specifically, claims regarding bacterially dominated nutrient cycling, fungal-mediated nutrient acquisition, or synergistic restoration potential remain hypothetical in the absence of direct functional measurements such as enzymatic assays, metagenomic profiling, or nutrient flux experiments. Deciphering such divergent patterns in microbial community assembly therefore provides a descriptive framework rather than a mechanistic proof of how plants influence belowground ecological processes. Understanding the interactions among the environment, plants, and soil microorganisms remains important for guiding future restoration research; however, any practical application of *P. anserina* as a restoration tool requires validation through replicated field trials, cost–benefit assessments, and functional assays before it can be recommended as a sustainable management strategy.

## 5 Conclusion

This study reveals the different impacts of *P. anserina* establishment on bacterial and fungal communities in bare patches of degraded alpine meadows on the Qinghai-Tibetan Plateau, enhancing our understanding of how plants affect microbial assembly processes in alpine meadow ecosystems. Bacterial community assembly is largely governed by deterministic environmental selection, whereas fungal communities are more strongly influenced by spatial processes and stochasticity. These different strategies are further reflected in network stability, with bacterial networks becoming looser due to the establishment of *P. anserina*, whereas fungal networks maintain stability through high modularity. To mitigate the rapid degradation of alpine meadows on the Qinghai-Tibetan Plateau under the pressures of global climate change and human factors, we tentatively recommend the targeted planting of *P. anserina* on small, completely bare degraded plots as a pilot-scale initial restoration measure, pending validation through replicated field trials and cost–benefit assessments. Future work should include the targeted isolation and functional characterization of fungal strains associated with *Preussia* and other potential keystone taxa, followed by manipulation experiments to test whether inoculation enhances grassland resilience. This study elucidates the microbial-level mechanisms underlying the restoration efficacy of *P. anserina* on bare patches, offering a preliminary mechanistic framework for bare-land ecological restoration. The insights from this study are consistent with global efforts to protect the Qinghai-Tibetan Plateau under the context of climate change, providing a “plant–soil microbe” centered framework for maintaining regional ecosystem functions and stability. Specific future directions include: (i) long-term field trials monitoring the persistence of the observed microbial shifts across multiple growing seasons; (ii) greenhouse and field inoculation experiments to validate the functional roles of the identified keystone taxa; (iii) comparative studies with other alpine pioneer species to determine whether the microbial mechanisms reported here are generalizable or species-specific; and (iv) integration of plant functional-trait data with metagenomic analyses to link microbial network topology to measurable ecosystem processes such as nutrient mineralization and soil aggregate stability.

## Data Availability

The data analyzed in this study are publicly available in the NCBI Sequence Read Archive (SRA) under BioProject accession number PRJNA1497275.
