# Supplementary material for: Potentilla anserina reshapes soil-microbe networks in alpine meadow restoration
Source: Front Microbiol. 2026 Aug 11;17:1890269. doi: 10.3389/fmicb.2026.1890269 (PMC13503552; doi:10.3389/fmicb.2026.1890269)
Supplement: Supplementary file 1 [file Data_Sheet_1.docx]

Table S1 Soil properties from different samples

|  | R | F | CK |
| --- | --- | --- | --- |
| WC% | 24.27±3.56a | 17.13±1.58a | 16.00±0.91a |
| BD (g.cm^-3^) | 0.67±0.04b | 0.81±0.04a | 0.92±0.07a |
| pH | 7.07±0.15b | 7.04±0.14b | 7.70±0.08a |
| SOM (g/100g) | 4.84±0.10a | 0.38±0.08c | 1.88±0.01b |
| TN (g kg–1) | 1.34±0.03a | 0.11±0.02c | 0.52±0.00b |
| TP (g kg–1) | 4.21±0.08a | 0.33±0.07c | 1.64±0.01b |
| MBC (mg kg–1) | 525.31±42.72a | 493.20±40.80a | 235.89±20.49b |
| MBN (mg kg–1) | 74.67±7.20a | 66.17±5.01a | 31.69±2.24b |

Different letters within each row indicate significant differences (*P* < 0.05) between zones.

Abbreviations: WC%, water content; BD, bulk density; SOM, soil organic matter; TN, total nitrogen; TP, total phosphorus; MBC, microbial biomass carbon; MBN, microbial biomass nitrogen.

Table S2 Principal component analysis of soil variables with eigenvalues and the cummulative proportion explained by the first three principal components (PC). The component correlation scores (eigenvectors) with loadings greater than |0.8| are bolded.

|  | PC1 | PC2 | PC3 |
| --- | --- | --- | --- |
| Eigenvalue | 3.928 | 2.042 | 0.959 |
| Proportion Explained | 49.095 | 25.529 | 11.99 |
| Cumulative proportion | 49.095 | 74.624 | 86.614 |
| WC% | 0.555 | 0.269 | -0.715 |
| BD | -0.735 | 0.144 | 0.437 |
| pH | -0.275 | 0.566 | 0.22 |
| SOM | **0.905** | 0.344 | 0.232 |
| TN | **0.904** | 0.35 | 0.228 |
| TP | **0.905** | 0.349 | 0.226 |
| MBC | 0.507 | **-0.822** | 0.095 |
| MBN | 0.539 | -0.768 | 0.203 |

Table S3 The PC values of sampling sites and probability values for the analysis of variance (ANOVA) of the differences between zones for each extracted PC.

|  |  | PC1 | PC2 |
| --- | --- | --- | --- |
| R | R1-1 | 1.24 | -1.42 |
|  | R1-2 | 2.69 | -2.39 |
|  | R1-3 | 1.97 | -0.28 |
|  | R2-1 | 3.26 | 0.55 |
|  | R2-2 | 2.43 | 0.49 |
|  | R2-3 | 2.79 | 1.14 |
|  | R3-1 | 2.09 | -0.22 |
|  | R3-2 | 2.03 | 0.97 |
|  | R3-3 | 1.52 | -1.17 |
|  | F1-1 | -1.47 | 0.35 |
| F | F1-2 | -1.43 | -0.24 |
|  | F1-3 | -2.52 | 0.4 |
|  | F2-1 | -1.01 | 2 |
|  | F2-2 | -0.65 | 2.07 |
|  | F2-3 | -1.24 | 1.45 |
|  | F3-1 | -1.6 | 0.63 |
|  | F3-2 | -0.78 | 2.31 |
|  | F3-3 | -2.17 | 0.7 |
| CK | CK-1 | -2.24 | -2.24 |
|  | CK-2 | -1.84 | -2.06 |
|  | CK-3 | -1.31 | -1.54 |
|  | CK-4 | -1.78 | -1.5 |
| ANOVA |  | PC1 | PC2 |
| R vs F |  | <.001 | 0.017 |
| R vs CK |  | <.001 | 0.028 |
| F vs CK |  | 0.306 | <.001 |

Table S4

|  | Bacterial communities | | Fungal communities | |
| --- | --- | --- | --- | --- |
|  | *F* | *P* | *F* | *P* |
| Sequencing dataset | **4.33** | **0.001** | **4.18** | **0.006** |
| R *vs.* F | 3.254 | 0.135 | **4.74** | **0.045** |
| R *vs.* CK | **6.201** | **0.009** | **6.47** | **0.027** |
| F *vs.* CK | **4.623** | **0.027** | 3.73 | 0.082 |

Fig. S1 The Rarefaction curves of the number of operational taxonomic units (OTUs) for soil bacterial (A) and fungal (B) communities. Each line represents a soil sample.

Fig. S2 The shared bacterial and fungal OTUs number among soils for bacteria (a) and fungi (b) in three groups.

Fig. S3 The principal coordinate analysis based on Bray–Curtis dissimilarity of soil bacterial (a) and fungal (b) OTUs.

Fig. S4 The relative abundance of the domain soil bacterial and fungal phyla.

**b**

|  | num.edges | num.vertices | connectance | average.degree | average.path.length | clustering.coefficient | diameter |
| --- | --- | --- | --- | --- | --- | --- | --- |
| R | 2487 | 369 | 0.036629551 | 13.4796748 | 6.035037736 | 0.635365233 | 15.42485101 |
| F | 1808 | 353 | 0.02910121 | 10.24362606 | 7.237173508 | 0.598795181 | 18.2994051 |
| CK | 2986 | 381 | 0.041248791 | 15.67454068 | 6.626666786 | 0.663530218 | 24.04944852 |

Fig. S5 Network of co-occurring bacterial OTUs based on a correlation analysis in the three zones (a). Nodes are colored according to phyla. The legend shows main phylum in order of their relative abundance. Topological properties of bacterial and fungal co-occurring networks (b). The OTUs shown in the figure represent keystone taxa.

**b**

|  | num.edges | num.vertices | connectance | average.degree | average.path.length | clustering.coefficient | diameter |
| --- | --- | --- | --- | --- | --- | --- | --- |
| R | 2137 | 210 | 0.0973798131692868 | 20.352380952381 | 6.47366957025209 | 0.84980379846178 | 20.2579712823914 |
| F | 2218 | 218 | 0.0937724601530461 | 20.348623853211 | 4.74215277991111 | 0.814701526622469 | 14.4274071645035 |
| CK | 1621 | 198 | 0.0831154181407989 | 16.3737373737374 | 7.70855332873128 | 0.84838011819333 | 20.2742808477438 |

Fig. S6 Network of co-occurring fungal OTUs based on a correlation analysis in the three zones (a). Nodes are colored according to phyla. The legend shows main phylum in order of their relative abundance. Topological properties of bacterial and fungal co-occurring networks (b). The OTUs shown in the figure represent keystone taxa.
